# Biplanar radiographic analysis of knee alignment: a stepwise approach for phenotype classification and knee arthroplasty planning

**DOI:** 10.1530/EOR-2024-0155

**Published:** 2025-10-01

**Authors:** Shuhei Hiyama, Reuben P Rao, Tsuneari Takahashi, Jeya Palan, Hemant Pandit

**Affiliations:** ^1^ Leeds Institute of Rheumatic and Musculoskeletal Medicine (LIRMM), University of Leeds, Leeds, UK; ^2^ Department of Orthopedics, Jichi Medical University, Shimotsuke, Japan; ^3^ Sarawak General Hospital, Kuching, Sarawak, Malaysia

**Keywords:** osteoarthritis, knee, knee joint, arthroplasty, replacement, lower extremity, postural balance

## Abstract

- This review presents a standardized, stepwise method for biplanar radiographic analysis of knee alignment, integrating both coronal and sagittal measurements for use in arthritic and non-arthritic knees.
- It critically compares leading classification systems, including the coronal plane alignment of the knee (CPAK) and the functional knee phenotype classifications. While CPAK provides a simplified 2D coronal model, the functional phenotype system offers a more granular, 3D approach that includes segmental deformities and has recently been expanded to incorporate laxity parameters.
- Sagittal plane parameters – including posterior tibial slope and femoral component flexion/extension – are essential for comprehensive alignment assessment. These factors influence implant positioning, knee kinematics, and postoperative function.
- The review outlines preferred measurement techniques, highlighting the value of long-leg weight-bearing radiographs and discussing the limitations and variability of 2D versus 3D imaging approaches.
- Incorporating both alignment and soft tissue behavior provides a more individualized approach to total knee arthroplasty planning and may lead to improved outcomes by better replicating native knee biomechanics.

## Introduction

Implantation of a well-aligned total knee arthroplasty (TKA) requires detailed preoperative planning, including a clinical assessment and analysis of radiographs. Various alignment philosophies are utilized in performing TKA, and over the past decade a personalized approach to TKA is increasingly followed (1, 2, 3). A systematic alignment approach has been defined as either to aim for a neutral mechanical alignment (MA) or an individualized approach to reproduce the native knee’s pre-arthritic alignment, either with or without pre-decided boundaries. Neutral MA of the knee is defined by a mechanical hip-knee-ankle angle of 0° ± 3° (4). It has been favored in TKA to achieve symmetrical loading of bearing surfaces, a straight leg with a horizontal joint line, and has a proven long-term implant survival (5). However, MA ignores the normal knee’s joint line obliquity (JLO) and variations in individual knee alignment, and has been blamed for a percentage of TKA patients being dissatisfied with prosthetic knees that feel ‘unnatural’. Studies suggest that natural neutral MA may be present in only approximately 15% of native knees (4, 6, 7, 8).

The TKA outcomes are in part related to the surgeon’s ability to reproduce the desired lower limb alignment and JLO restoration. This has spurred a growing emphasis on restoring patient-specific pre-arthritic knee alignment and, with this, the development of knee alignment classifications such as the functional knee phenotype classification (9), the coronal plane alignment of the knee (CPAK) classification (4), and the coronal extra-articular deformity phenotype (CEDP) classification (10). These observe precise phenotypical divisions, and their development has highlighted the need for standardization and systematization in the radiographic analysis of knee alignment.

Various terminologies and techniques have been described to measure limb and joint alignment, often originating from the literature on limb deformity correction. To date, all classification systems have been based on plain radiographs, which are two-dimensional (2D) representations of three-dimensional (3D) anatomical structures. However, these images can be misleading due to observer variability, technical limitations (radiographs are not always perfectly standardized), subtle anatomical differences between individuals, and rotational or projectional variations. Recent reviews have furthermore highlighted the substantial variability in methods used for 3D leg alignment analysis, underscoring the lack of consensus on how to derive axes and joint orientations from 3D bone models (11). This prevents the establishment of universal reference values and makes it difficult to compare alignment parameters across studies (12, 13, 14).

This review aims to introduce a standardized sequence of radiographic measurements that are applicable to both arthritic and non-arthritic knees and align with the requirements of most current classification systems. While numerous measurement techniques and terminologies exist, inconsistency and lack of clarity often lead to confusion. Inaccurate measurements can result in malalignment and poor surgical outcomes. This review provides a practical guide, highlighting common pitfalls and offering strategies to minimize errors. What makes this approach original is its integrative nature – it synthesizes existing nomenclatures and techniques into a clear, applicable framework. In addition, by incorporating insights from recent 3D alignment studies (14), it places traditional 2D assessments in the context of modern alignment concepts.

## Measurement methodology

Table 1 contains a compilation of measurements from different classification systems, along with their definitions.

**Table 1:** Alignment parameters.

Measurement	Radiograph	Definition
Femoral mechanical axis	APLLR	Line connecting the center of the femoral head and the apex of the femoral intercondylar notch
Tibial mechanical axis	APLLR	Line connecting the midpoint of the tibial interspinous groove and the center of the talus
mHKA	APLLR	Acute angle between the femoral mechanical axis and the tibial mechanical axis at the point of intersection (negative value for varus and positive value for valgus)
Distal femur joint orientation line	APLLR	Line connecting the most distal points of the medial and lateral femoral condyles
mLDFA	APLLR	Lateral angle formed between the femoral mechanical axis and the distal femur joint orientation line
Proximal tibial joint orientation line	APLLR	Line best fitted to the superior surface of the subchondral bone plate of the medial and lateral tibial plateaus
mMPTA	APLLR	Medial angle formed between the tibial mechanical axis and the proximal tibia joint orientation line
JLO = mMPTA + mLDFA	APLLR	The obtuse angle formed between the knee joint line and the floor in a standing AP radiograph
Joint line convergence angle	APLLR	The acute angle formed between the distal femoral and proximal tibial joint orientation lines (negative value for medial apex and positive value for lateral apex)
LDFA	LLLR	Line connecting the centers of two circles, which are simultaneously tangent to the anterior and posterior cortices of the femur. The proximal circle is positioned just distal to the junction between the middle and distal thirds of the diaphysis, while the distal circle is positioned at the distal metadiaphyseal junction
Lateral distal femoral joint orientation line	LLLR	Line connecting the points where the femoral condyles join the anterior and posterior distal femoral metaphysis
Anatomical posterior distal femoral angle	LLLR	Posterior angle formed between the lateral distal femoral anatomical axis and the lateral distal femoral joint orientation line
Lateral proximal tibial joint orientation line	LLLR	Tangent to the deepest point of the medial plateau concavity
Lateral tibial central anatomical axis	LLLR	Line connecting the centers of two circles that are simultaneously tangent to the anterior and posterior tibial cortices. The proximal circle is positioned just distal to the tibial tuberosity, and the distal circle is positioned just proximal to the distal metaphyseal flare
PTS	LLLR	Acute angle formed between the perpendicular to the lateral tibial central anatomical axis and the lateral proximal tibial joint orientation line

APLLR, anteroposterior lower limb radiograph including hip, knee, and ankle joints; LLLR, lateral view lower limb radiograph including hip, knee, and ankle joints; mHKA, mechanical hip-knee-ankle angle; mMPTA, mechanical medial proximal tibial angle; JLO, joint line obliquity; mLDFA, mechanical lateral distal femoral angle; LDFA, lateral distal femur anatomical axis; PTS, posterior tibial slope.

### Coronal plane alignment

#### Radiographic assessment

Accurate and reproducible measurements of coronal alignment are crucial in the evaluation and surgical management of the knee, particularly in osteotomy and TKA (15). Weight-bearing conditions significantly affect the assessment of lower limb coronal alignment (16). The true MA can only be evaluated in long-leg radiographs when the limb is fully extended and bearing full weight on both legs under physiological loading (16). All coronal plane measurements are performed on a frontal plane anteroposterior lower limb radiograph (APLLR), as described by Paley (17), which includes the hip, knee, and ankle joints, with the knee in full extension and the patient weight-bearing in a bipedal stance. William (18) compared coronal alignment using a standardized radiographic protocol in single-leg, double-leg, and supine positions, noting that coronal deformity progressively increases from supine to double-leg and single-leg stances as weight-bearing load increases (19). The average difference between the supine and double-leg stance positions is 1.76°.

**Figure 1:**
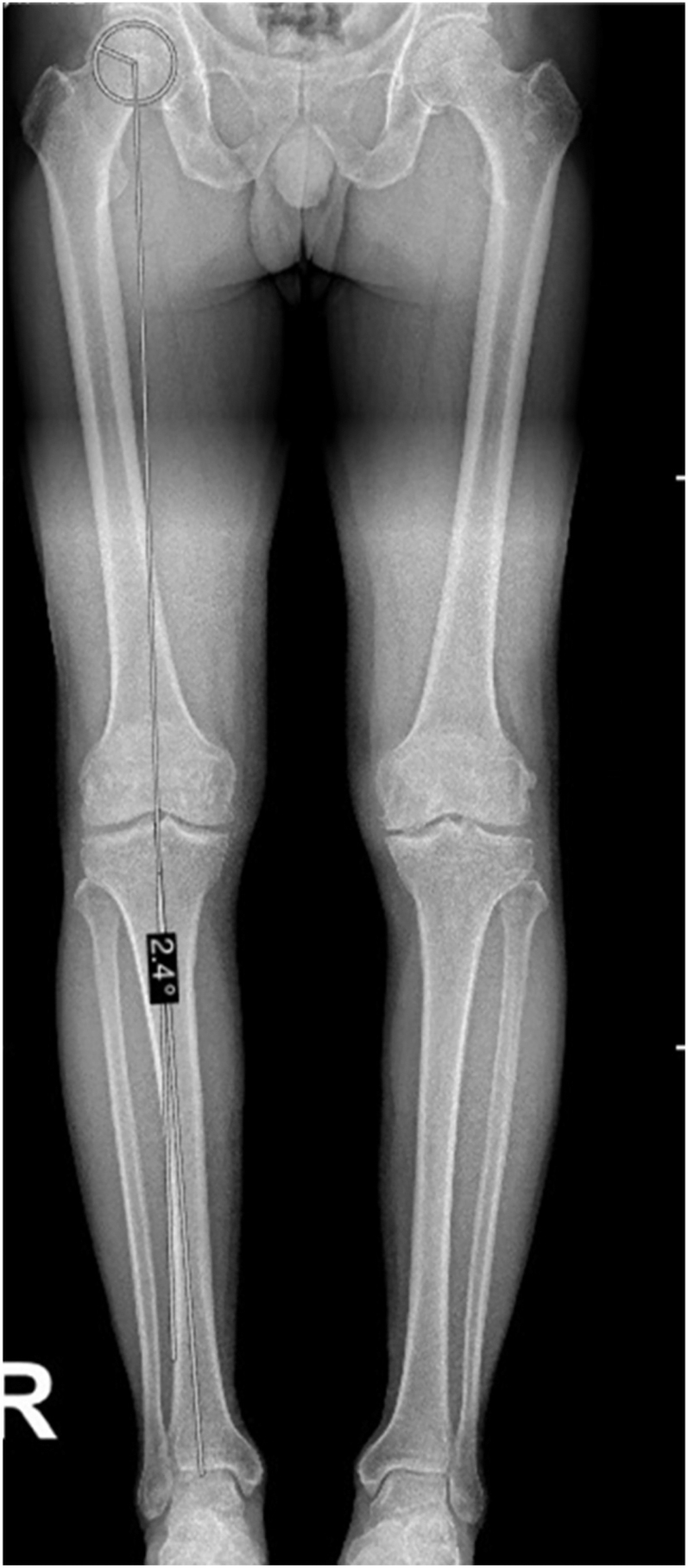
Measurement of mechanical hip-knee-ankle angle (mHKA) between mechanical axes of the femur and tibia.

Mechanical hip-knee-ankle angle (mHKA) is defined as the angle between the mechanical axes of the femur and tibia, with a deviation from 180° representing malalignment (Fig. 1). Negative values indicate varus, while positive values indicate valgus alignment (20). The proximal reference point of the femoral mechanical axis is the femoral head center, identified as the center of a circle fitted to the intact portion of the femoral head and/or acetabulum. This can be challenging in the presence of bone loss or deformity. In such cases, the circle is aligned to the preserved inferior portion of the femoral head or the medial acetabular wall (21). The apex of the femoral intercondylar notch (22) is considered the distal point of the femoral MA, while the center of the tibial interspinous groove is considered the proximal endpoint of the tibial MA (23). The center of the talus is taken as the distal end.

Deviation from constitutional mHKA may be caused by altered joint line convergence angle (JLCA), extra- or intra-articular tibial or femoral deformity, or knee joint subluxation. In comparison, the arithmetic hip-knee-ankle angle (aHKA), as described by Macdessi (24), is calculated from the mechanical lateral distal femoral angle (mLDFA) and the mechanical medial proximal tibial angle (mMPTA). As neither of these angular measurements ‘cross’ the knee joint, aHKA is unaffected by the changes in JLCA but may still be affected by extra-articular segmental deformity (10). Indeed, the accuracy of JLCA and/or HKA can be affected by significant bone loss associated with arthritis. A significant difference and a weak correlation between the values of the HKA and aHKA measures in the same subject were observed. The two analysis techniques provide different information, and their correlation is only partial (25). As such, either mHKA or aHKA is able to identify the presence but not the source of lower extremity malalignment (24, 26).

**Figure 2:**
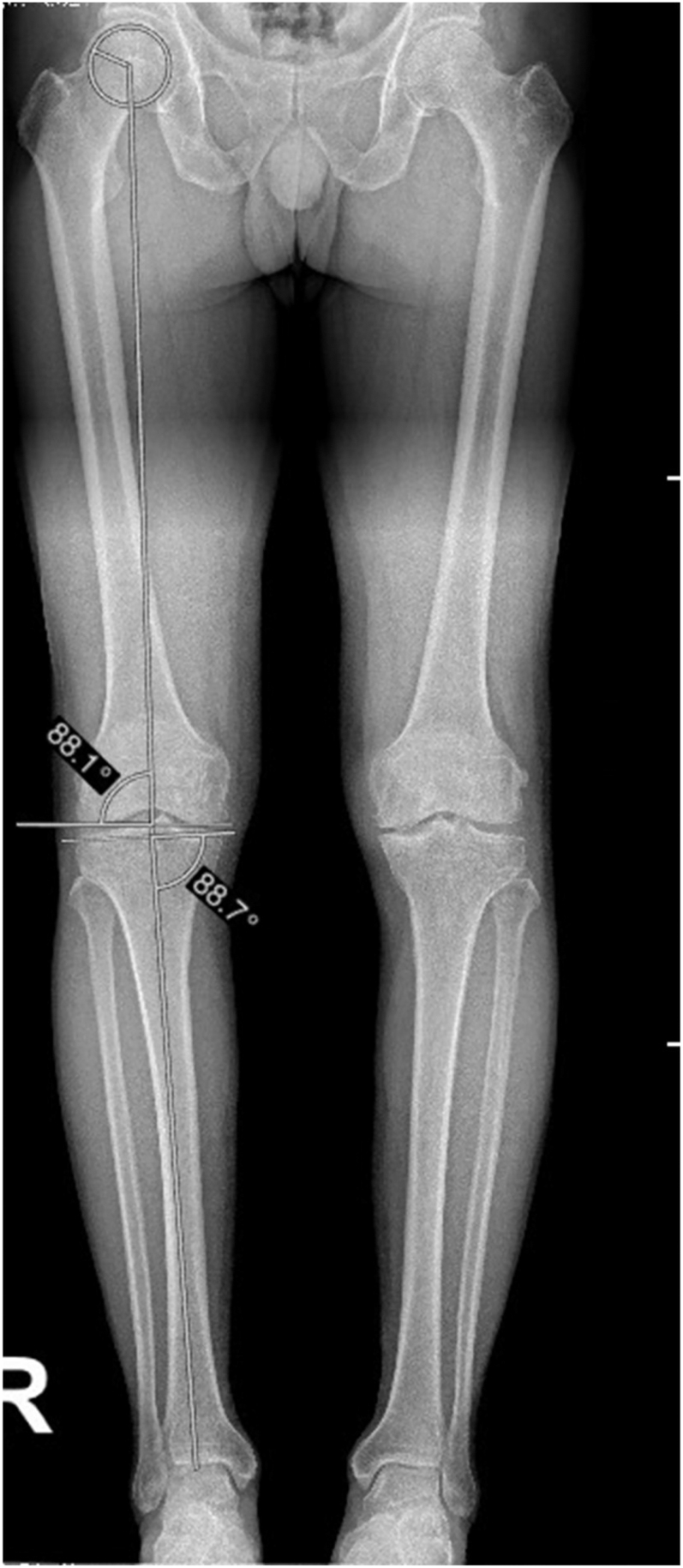
Measurements of mechanical lateral distal femoral angle (mLDFA) and mechanical medial proximal tibial angle (mMPTA).

Isolation and quantification of knee malalignment require the establishment of normal values for joint orientation angles (27). The stepwise malalignment test designed by Paley *et al.* (17) utilizes the same two measurements used to derive aHKA, with the added definition of a normal range (85°–90°) for both mLDFA and mMPTA. An mLDFA measurement outside this range is indicative of a femoral coronal plane deformity (<85°: valgus, >90°: varus), while an mMPTA measurement outside this range is indicative of a tibial coronal plane deformity (<85°: varus, >90°: valgus). The mLDFA is the lateral angle formed between the mechanical axis of the femur and the AP distal femur joint orientation line. The AP distal femur joint orientation line connects the most distal points of the medial and lateral femoral condyles. The mMPTA is the medial angle formed between the tibial MA and the AP proximal tibia joint orientation line (Fig. 2).

JLCA is the acute angle formed between the AP distal femoral and proximal tibial joint orientation lines. Altered JLCA is common in knee arthritis (20, 28) and leads to altered mHKA. It is calculated as JLCA = mHKA – aHKA. A negative value indicates a medial apex JLCA (wider lateral joint space), while a positive value indicates a lateral apex JLCA (wider medial joint space). Normal JLCA ranges from 0° to −3° (29).

Medio-lateral subluxation of the tibiofemoral joint will cause deviation of lower extremity MA and alter mHKA. To detect subluxation, the femoral and tibial MAs are drawn from separate points in the knee (27).

### Coronal alignment phenotypes

CPAK classification introduced by MacDessi *et al.* (4) categorizes native knee phenotypes based on aHKA and JLO, and has nine phenotypes from combinations of varus/neutral/valgus aHKA and distal/neutral/proximal JLO. The CPAK system classifies (4) knees into nine theoretical types using two coronal plane variables: aHKA and JLO, and dividing each variable into three possible ranges (aHKA: varus/neutral/valgus; JLO: apex distal, neutral, apex proximal). Type II was the most common, followed by type I, for both groups, healthy knees and osteoarthritic knees, and independent of ethnicity. In both groups, a neutral mechanical axis with a joint line that is perpendicular to this axis had a frequency of ∼15%. Although initially appealing for its structured approach, CPAK has notable limitations. CPAK is a 2D system and does not capture the complexity of the 3D alignment or segmental deformities. Şahbat *et al.* reported that CPAK correctly identified the joint line apex in less than half of the cases (30). Loddo *et al.* demonstrated that CPAK cannot differentiate between segmental deformities in the femur and tibia (31). These findings collectively question the clinical value of CPAK, especially in surgical planning and outcome prediction (32). To address the limitations of CPAK, Hirschmann *et al.* proposed the functional knee phenotype classification (8), incorporating three parameters: HKA, femoral mechanical angle, and tibial mechanical angle. Each is categorized as varus, neutral, or valgus, allowing for up to 125 possible phenotypes that provide detailed insights into segmental contributions to malalignment. Recent studies support the clinical relevance of the functional classification. Chelli *et al.* demonstrated sex-based differences in femoral and tibial alignment using functional phenotypes (33). Kobayashi *et al.* found that the system helped preserve native alignment and avoid excessive bone resection in Japanese patients undergoing restricted kinematic alignment TKA (34). Liu *et al.* showed that functional phenotypes better represent anatomical variability in Chinese knees, where CPAK failed to distinguish femoral varus cases adequately (35).

The functional knee phenotype classification is based on three different coronal plane variables, each of which is divided into five ranges. In Hirschmann’s series of young, non-arthritic individuals, out of the 125 theoretically possible combinations, 43 distinct phenotypes were observed (9). The most common phenotype among men and women was NEUHKA0° + NEUFMA0° + NEUTMA0°, while two-thirds of the population comprised the eight most common phenotypes (9). Recent large-scale studies have refined the definition of coronal knee alignment in the context of TKA, classifying it into normal, neutral, deviant, and aberrant types based on population-based data (8). These findings support the need for individualized alignment strategies rather than relying solely on traditional MA targets. Furthermore, gender-based differences in functional knee phenotypes have been demonstrated, suggesting that sex-specific considerations may also be important in alignment assessment and surgical planning (36).

The CEDP also divides knees into nine groups based on the CPAK system, but considers femoral (LDFA) and tibial (MPTA) segmental deformities separately. The three most common types, in order, were types B, A, and E, which correspond to CPAK types II, I, and V. To achieve precision, surgeons should incorporate individual MPTA and mLDFA values, ensuring the accurate identification and replication of segmental coronal deformities in knee surgery (10).

Overall, the functional phenotype classification correlates better with actual bony morphology, provides greater reproducibility, and can be applied across diverse populations. Its finer granularity and anatomical specificity make it more suitable for patient-specific surgical planning. By contrast, while CPAK offers a simplified framework, its limitations in precision, segmental analysis, and clinical applicability render it less ideal for contemporary alignment strategies.

### Sagittal plane alignment

All measurements are taken on lateral long-leg radiographs (LLLRs) visualizing the extremity from hip to ankle joint. To achieve this, the patient should be placed with the flexion angle of the target limb parallel to the detector, with the knee fully extended and the opposite hemipelvis externally rotated (37).

### Femoral measurements

**Figure 3:**
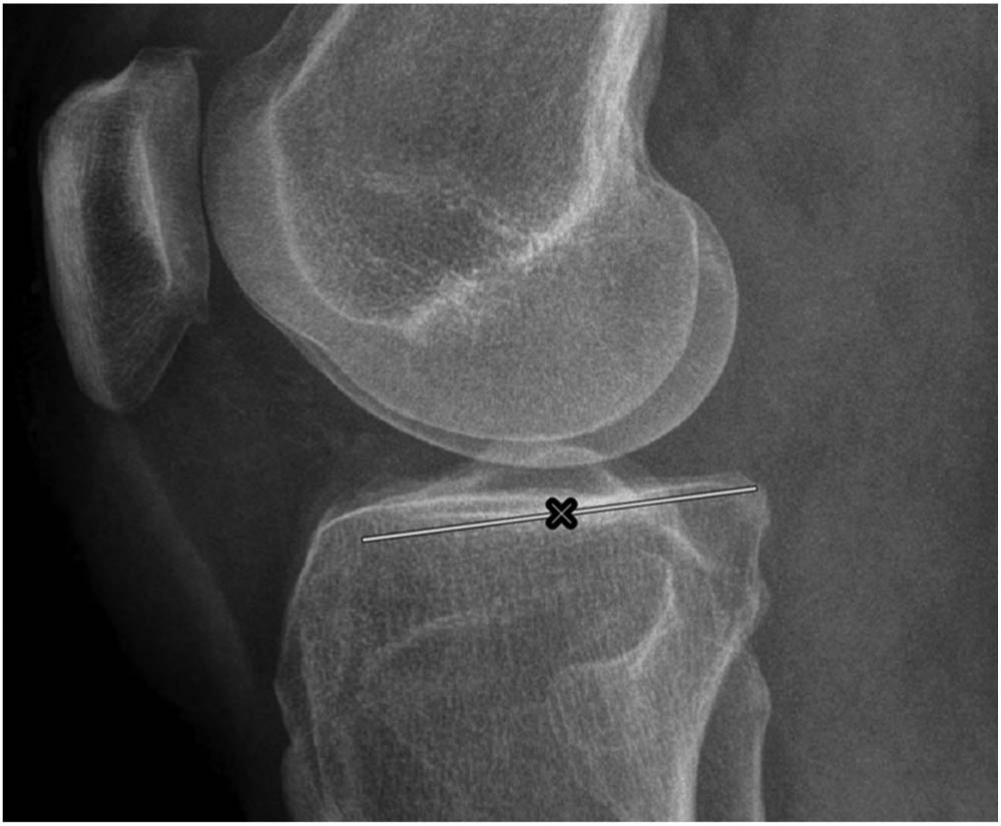
Proximal tibial joint orientation line. The cross marks the deepest point of the medial plateau concavity. The line is tangent to the curve at the deepest point.

In the femur, the lateral long axis is represented by the lateral distal femur anatomical axis (DFAA). Anatomical posterior distal femoral angle (aPDFA) is formed between the DFAA and lateral distal femoral joint orientation line. The DFAA is drawn by connecting the centers of two circles. The proximal circle is positioned just distal to the junction between the middle and distal thirds of the diaphysis, while the distal circle is drawn at the distal metadiaphyseal junction. Both circles are drawn so that they are simultaneously tangent to the anterior and posterior cortices. The lateral distal femoral joint orientation line is drawn connecting the points where the femoral condyles join the anterior and posterior distal femoral metaphysis on a lateral view radiograph of the knee (Fig. 3). The normal range for aPDFA is 79°–87°.

### Tibial measurements

The tibial aspect of the sagittal plane consists of the posterior tibial slope (PTS). This measurement may be the most difficult to reliably and accurately measure, as the reported methods for measuring PTS are numerous and significantly different. Such heterogeneity stems from varied definitions of the proximal tibial joint orientation line, varied representations of the lateral long axis of the tibia, and the use of different radiographic images. Tibial joint orientation line can be difficult to identify accurately due to the superimposition of medial and lateral tibial plateaus on lateral radiographs. A commonly used method for defining the joint orientation line is to draw a line connecting the anterior and posterior margins of the tibial plateau (38, 39). In arthritic knees, this task is made more difficult by the presence of anterior and posterior marginal osteophytes and/or associated bone loss. A reported method (40), possibly more applicable in the arthritic knee, is to draw a tangent to the deepest point of the medial plateau concavity (Fig. 3).

**Figure 4:**
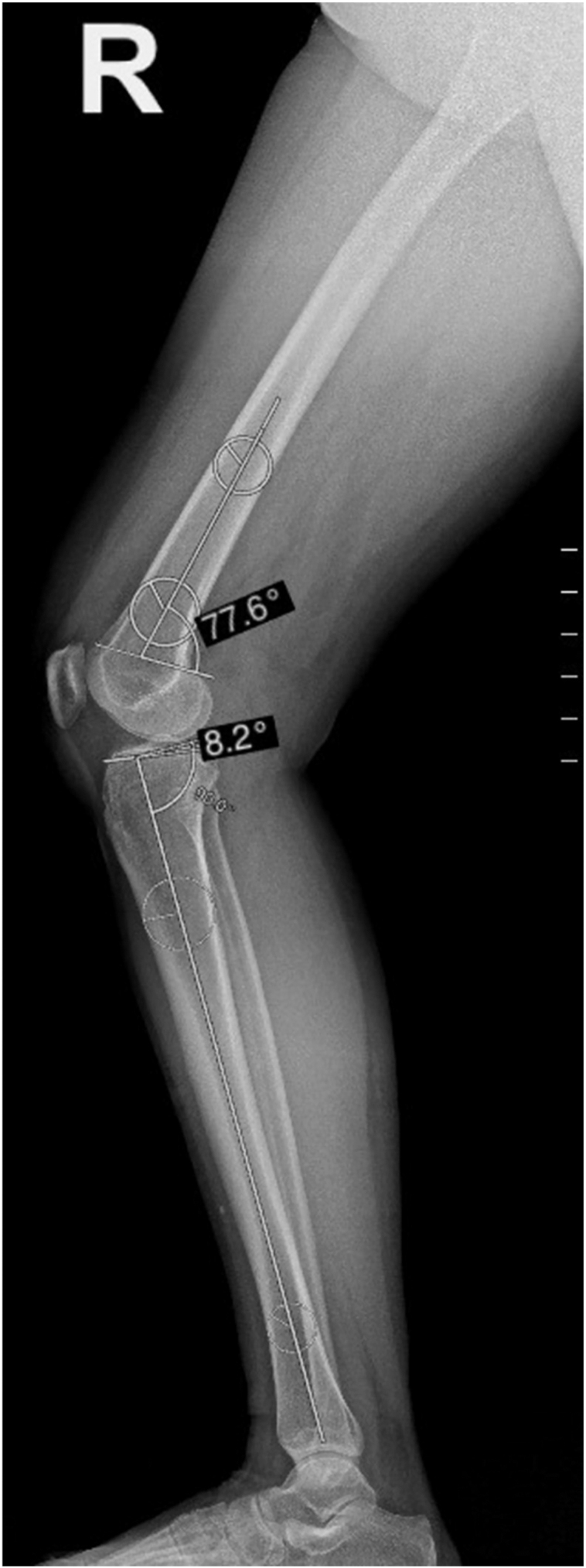
Measurements of anatomical posterior distal femoral angle and PTS.

Among the axes used to represent the lateral long axis of the tibia are the modified mechanical axis, anterior cortical line (ACC), posterior cortical line (PCC), proximal anatomical axis (PAA), central anatomical axis (CAA), and fibular shaft axis (41). The CAA is the mid-diaphyseal axis of the entire tibial diaphysis. It is drawn by connecting the centers of two circles that are simultaneously tangent to the anterior and posterior cortices. The proximal circle is drawn just distal to the tibial tuberosity, while the distal circle is drawn just proximal to the distal metaphyseal flare (42). Compared to ACC, PCC, and PAA, the CAA does not rely on accurate radiographic magnification because it does not involve measurements of shaft length to position the necessary mid-diaphyseal points. It can also be reproduced intraoperatively by using a tibial intramedullary guide. The PTS is the angle formed between the perpendicular to the CAA and the lateral proximal joint orientation line of the tibia (Fig. 4). The average range of PTS measured using CAA is 15.9° ± 3.7° (42). However, variations in PTS exist based on factors such as age, gender, race, and region.

Short knee radiographs are commonly used for measurement of PTS. However, these measurements may not be truly representative, especially in the presence of significant sagittal bowing (43). Hees *et al.* found that as anterior tibial bowing increases, the PTS measured on such radiographs is underestimated in comparison to the slope measured along the lateral mechanical axis (43). It is recommended that PTS be measured on LLLRs. Garra highlighted the effect of tibial length on radiographic PTS measurements, concluding that both the accuracy and precision of these measurements improved as the length of the tibia used to define the anatomical axis increased (44). In addition, the medial PTS (MPTS) and lateral PTS (LPTS) differ within the same knee, with MPTS generally being higher than LPTS. Calek demonstrated substantial inter- and intra-individual variability in MPTS and LPTS, noting that conventional single-slope resection techniques in TKA fail to reproduce native slope geometry in most cases. In their CT-based study of 301 healthy knees, more than 40% showed a difference of over 3° between MPTS and LPTS, and only 15% of knees had both slopes fall within the conventional target range for PTS (45).

In addition, Sadoghi *et al.* highlighted that constitutional sagittal alignment is often underappreciated in TKA planning. They argued that fixed target slopes and component positioning may alter a patient’s natural kinematics, and suggested individualized, patient-specific strategies for optimizing TKA outcomes (46).

Several investigators have studied potential differences in PTS among sexes or ethnic groups. Hashemi (47) reported that females exhibited higher MPTS and LPTS compared to males, a finding later confirmed by Weinberg (48). Conversely, Pangaud (49) found that males had a significantly higher LPTS than their female counterparts, and no difference was seen between sexes in MPTS (50). Bryce (50) reported that African Americans/Blacks and Asian Americans have a greater PTS compared to Whites. Nearly 25% of individuals have clinically significant PTS of <6° or >12°, with no difference in PTS among age or sex groups.

Zachary *et al.* (51) aimed to analyze the impact of preoperative PTS and CPAK on pre- and postoperative knee kinematics following PCL-conserving TKA. Their findings (51) indicate notable differences between type A (≤8°) and type B (>8°) PTS knees. Specifically, type B knees exhibited greater knee flexion both before and after surgery. Although patients with type A knees showed more substantial improvements in knee flexion following TKA, they still had significantly less flexion postoperatively. Despite greater improvements in type A knees, they remained less flexible pre- and post-surgery compared to type B knees. Therefore, PTS is a crucial variable that may help predict postoperative knee function.

### Stepwise assessment of arthritic knee alignment

#### Step 1

Draw femoral and tibial mechanical axes. Extend femoral mechanical axis distally and tibial mechanical axis proximally. If the axes do not intersect at the tibial interspinous groove, this is indicative of mediolateral knee joint subluxation. Measure the acute angle between the two axes. This is the mHKA. By convention, a varus angle is represented by a negative value, while a valgus angle is represented by a positive value. Measured range of mHKA in young healthy adults is −1.5°–1.5° (9).

#### Step 2

Draw the distal femoral and proximal tibial joint orientation lines and analyze the femoral condyles and tibial plateaus for the presence of bony deformities as described before. The presence of intra-articular bony deformities precludes reliable assessment of constitutional alignment. In the absence of these deformities, measure mLDFA and mMPTA.

#### Step 3

Calculate aHKA and JLO based on formulas; aHKA = mMPTA – mLDFA, and JLO = mMPTA + mLDFA (4). JLCA can be measured between joint orientation lines of the femur and tibia or derived from the formula; JLCA = mHKA – aHKA. A negative value indicates a medial apex JLCA (wider lateral joint space), while a positive value indicates a lateral apex JLCA (wider medial joint space).

#### Step 4

Compare mLDFA and mMPTA to normal range: 85°–90°, respectively (17). A deranged mLDFA is suggestive of a femoral coronal plane deformity, while a deranged mMPTA is suggestive of a tibial coronal plane deformity.

#### Step 5

Sagittal alignment of the knee is determined by measuring aPDFA and PTS. A measured aPDFA outside the normal range of 79°–87° is indicative of femoral sagittal plane deformity (<79°: procurvatum, >87°: recurvatum). A measured PTS outside the normal range of 12.2°–19.6° is indicative of tibial sagittal plane deformity.

### The role of laxity

Bony alignment alone does not fully capture the complexity of knee mechanics (52). Ligamentous laxity, part of the soft tissue envelope, plays a crucial role in achieving proper knee balance and function. Therefore, it must be considered alongside alignment in preoperative planning and classification. Recent studies, particularly by Grosso, have shown that knees with similar coronal bony alignment can exhibit widely varying medial and lateral soft tissue stiffness during intraoperative distraction, especially in varus knees undergoing TKA. This variability suggests that bone morphology alone cannot reliably predict soft tissue behavior, challenging the notion that restoring constitutional alignment will automatically lead to balanced tension (53). As such, alignment should be viewed as only one aspect of knee reconstruction. A comprehensive strategy should integrate both the static geometry of the bones and the dynamic characteristics of soft tissue. Future developments in TKA planning may benefit from incorporating intraoperative laxity assessments – potentially via robotic or sensor-assisted technologies – into alignment algorithms (54).

## Discussion and summary

This comprehensive review offers a systematic and clearly defined approach to the coronal and sagittal analysis of constitutional alignment of the native knee. The techniques and elements in this system are specifically compiled to be compatible for assessing radiographs of patients with knee arthritis.

The functional knee phenotype system (10) was the first to draw attention to the complexity and diversity of constitutional knee alignment. It introduced the concept of combining limb and knee alignment parameters into a complex but systematic nomenclature and precisely divided knees into multiple groups based on mean and standard deviation values. The CPAK system (4) is useful for analyzing constitutional coronal alignment of the arthritic knee as it eliminates the influence of asymmetrical joint space on the hip-knee-ankle angle. The inherent pragmatism and clear terminology of this system facilitate communication and comparison of results. More recently, the CEDP system, built on the strength of the CPAK grid system, incorporates separate analyses of femoral and tibial extra-articular deformities. It also emphasizes the advantages of using the ‘anatomical range’ of mLDFA and mMPTA (87.0° ± 2°) for more precise separation of phenotypes. Each of these systems offers valuable insights into the diversity of constitutional knee alignment with the common goal of enabling accurate restoration of native alignment in knee arthritis.

Indeed, some studies have reported that the differences between OA and non-OA knees are small regarding coronal femoral and tibial joint line orientation, whereas other studies have stated that there is a wide variation in coronal alignment in both arthritic and non-arthritic knees (55, 56, 57). Hess (58) conducted a systematic literature review to examine the variability in coronal femoral and tibial alignment in patients with arthritic knees. This review not only highlights the variation in the coronal alignment but also demonstrates the variability with which different parameters are reported. Similarly, there is considerable variation in PTS, differences in slope between the medial and lateral tibial plateaus, as well as variability in trochlear geometry. Hazratwala (59) analyzed 4,116 knee CTs from the 360 Knee Systems© database, focusing on arthritic pre-operative TKA patients. The medial and lateral PTS ranged from 5° anterior to 25° posterior. A differential PTS of more than 5° between the medial and lateral sides was found in 22.6% of patients. Of these, 14.5% exhibited a greater lateral PTS, with a mean difference from the medial PTS of 4.8° ± 5.0°, while 31.0% had a greater medial PTS, with a mean difference from the lateral PTS of 5.7° ± 3.2°. In addition, 14% of trochlear angles (TA) relative to the distal femoral angle and 5.2% of TA relative to the posterior condylar angle varied by more than 10°.

All these systems, however, share common limitations. First, the elements of analysis are poorly defined. Most measurements are only briefly and/or vaguely described, leaving room for interpretation. This can lead to a lack of uniformity and accuracy in analysis and classification. Second, some of the measurement parameters used are prone to influence by arthritic changes of the knee. As described above, these aberrations may decrease the accuracy of measurements and be unrepresentative of constitutional alignment. Finally, all the previously mentioned classification systems are purely based on coronal plane parameters and make no attempt to incorporate the sagittal parameters of constitutional alignment. Assessment of sagittal plane JLO, i.e., PTS, is equally important. Changes in PTS, whether by increasing or decreasing it, could significantly impact PCL function. An increase in PTS may lead to greater strain and overload on the collateral ligaments, causing abnormal forces at the implant–bone interface, which may result in instability or excessive wear (60, 61). On the other hand, a reduction in PTS may compromise knee stability by altering load distribution and potentially diminishing the PCL’s capacity to provide posterior stability (60, 61). While the gold standard for measuring PTS is through CT or MRI, or at least lateral full tibia length radiographs, these are not always readily available (62). The tibial axis, defined by the midpoint of the tibial plateau and the midpoint of the ankle joint, serves as a reliable reference for measuring PTS, but determining it intraoperatively can sometimes be challenging. The anterior tibial cortex is often used as an additional reference. Other proposed reference axes for measuring tibial slope include the posterior tibial cortex, the fibula axis, or the line connecting the midpoints of the medullary canal at two different levels (circle method) (41, 63). There is a need for a simple method of measuring PTS that is accurate and reproducible.

If the common goal is for knees to be analyzed and restored reliably, then the sagittal parameters of constitutional alignment are equally important to define and analyze. Therefore, a system of well-defined, biplanar, systematic analysis of knee alignment is needed to ensure precise and comparable results.

### Limitations

The literature shows significant heterogeneity in alignment definitions and measurement methods, making comparisons difficult. Second, newer classification systems (CPAK, functional phenotypes, CEDP) are based mainly on cross-sectional data, with limited evidence of clinical utility or long-term outcomes. Third, the review focuses only on coronal and sagittal alignment, excluding axial rotation and dynamic factors such as soft tissue laxity. Finally, conclusions may inherit biases from the included studies. Nonetheless, evidence supports a shift toward a more holistic, biplanar alignment framework to improve knee arthroplasty outcomes.

## Conclusion

We have compiled and outlined a stepwise sequence of clearly defined coronal and sagittal alignment measurements of the knee that can be applied to most existing classification systems. These measurements were specifically selected to mitigate the influence of knee arthritis on alignment analysis and for their intraoperative reproducibility in TKA. Such standardization will aid surgeons in restoring patient-specific alignment more accurately and pave the way for future studies to validate and refine this framework.

## ICMJE Statement of Interest

The authors declared the following potential conflicts of interest with respect to the research, authorship, and/or publication of this article: one of the authors received a grant for another clinical research as a principal investigator from MicroPort Orthopedics, Inc., Arlington, TN, USA.

## Funding Statement

HP and JP are supported in part by the National Institute for Health and Care Research (NIHR) Leeds Biomedical Research Centre (BRC) (NIHR203331). The views expressed are those of the author(s) and not necessarily those of the NHS, the NIHR or the Department of Health and Social Care.
